# RNAi Screen Indicates Widespread Biological Function for Human Natural Antisense Transcripts

**DOI:** 10.1371/journal.pone.0013177

**Published:** 2010-10-04

**Authors:** Mohammad Ali Faghihi, Jannet Kocerha, Farzaneh Modarresi, Pär G. Engström, Alistair M. Chalk, Shaun P. Brothers, Eric Koesema, Georges St. Laurent, Claes Wahlestedt

**Affiliations:** 1 Department of Neuroscience, The Scripps Research Institute, Jupiter, Florida, United States of America; 2 Department of Molecular Therapeutics, The Scripps Research Institute, Jupiter, Florida, United States of America; 3 Bioinformatics Institute, Hinxton, Cambridge, United Kingdom; 4 National Centre for Adult Stem Cell Research, Eskitis Institute for Cell and Molecular Therapies, Griffith University, Brisbane, Australia; 5 Department of Molecular Biology, Cell Biology and Biochemistry, Brown University, Providence, Rhode Island, United States of America; 6 Immunovirology-Biogenisis Group, University of Antioquia, Medellin, Colombia; Agency for Science, Technology and Research (A*STAR), Singapore

## Abstract

Natural antisense transcripts represent a class of regulatory RNA molecules, which are characterized by their complementary sequence to another RNA transcript. Extensive sequencing efforts suggest that natural antisense transcripts are prevalent throughout the mammalian genome; however, their biological significance has not been well defined. We performed a loss-of-function RNA interference (RNAi) screen, which targeted 797 evolutionary conserved antisense transcripts, and found evidence for a regulatory role for a number of natural antisense transcripts. Specifically, we found that natural antisense transcripts for CCPG1 and RAPGEF3 may functionally disrupt signaling pathways and corresponding biological phenotypes, such as cell viability, either independently or in parallel with the corresponding sense transcript. Our results show that the large-scale siRNA screen can be applied to evaluate natural antisense transcript modulation of fundamental cellular events.

## Introduction

Since the publication of the human genome, estimates of the number of protein coding genes have remained remarkably stable. In contrast, many surprises have come from the study of non-protein-coding regions, challenging our understanding of mammalian gene expression. Several high-throughput transcriptomic efforts have demonstrated that a vast majority of the mammalian genome is transcribed *in vivo* [1], [2], [3], [4], [5], [6]. When measured as a percentage of the total genome, non-protein-coding RNAs (ncRNAs) increase consistently with developmental complexity of organisms across the entire range of evolution [7]. In humans, ncRNAs comprise a majority of the transcriptional output [8], [9], [10]. While this ncRNA transcriptional output spans an impressive range, from the very short to over 100 kb, most of the longer transcripts have received far less experimental scrutiny compared to the small ncRNAs.

A sequencing-based transcriptomics effort, conducted by the FANTOM3 consortium, confirmed and substantially extended the previously existing reports [11], [12], [13], [14] on the intriguing family of long ncRNAs called natural antisense transcripts (NATs) [3], [15]. Natural antisense transcripts are RNA molecules which are transcribed from the opposite DNA strand to other transcripts and overlap in part with sense RNA, promoter or regulatory region. Both sense and antisense RNAs can encode proteins or be non-protein-coding transcripts; however, the most prominent form of antisense transcription in the mammalian genome is a non-protein-coding antisense RNA partner of a protein-coding transcript [15]. The antisense RNAs are often transcribed from the same genomic locus as the sense transcript (*cis*-NATs), which are focus of the current study. Sense and antisense RNAs can also be transcribed from distinct genomic loci (*trans*-NATs). The FANTOM3 collection includes at least 1,000 sense-antisense transcript pairs, well conserved between mouse and human, and many thousands of non-conserved pairs [16]. While some NATs code for proteins, the majority lack conventional open reading frames and therefore represent ncRNA [3]. The functions of long ncRNA transcripts remain largely unknown [17]; nevertheless, there are a few examples of long ncRNAs inducing rapid changes in target gene expression during cellular responses to various forms of stress (*BACE1*-AS [18]; HSR [19]; HIF1a-AS [20]; NRON [21]). Stress in the nervous system may represent a focus of activity for long ncRNAs, as the Allen Brain Atlas [22] revealed nearly one thousand of these species localized to highly specific cell type distributions in the mammalian brain [23]. Establishing the connection of long ncRNAs to chronic stress and the onset of neurodegeneration, we reported recently a NAT ncRNA that functions to stabilize *BACE1* mRNA and increase *BACE1* gene expression *in vivo* and *in vitro* [18]. Despite these and other examples of functional NATs [24], [25], it is still unclear whether these reports represent exceptions or if they describe more generalized regulatory architectures. Therefore, controversy still surrounds the fundamental and largely unanswered question of whether NATs participate in biologically significant information processing and regulation of macromolecular machineries.

The combination of traditional cell culture techniques with modern high-throughput genomics technology has enabled the simultaneous interrogation of thousands of genes, facilitating the functional analysis of complex biological processes [26], [27]. Among these, RNA interference (RNAi) can be used to expedite genome-wide loss-of-function (LOF) screens in mammalian systems [26], [27], [28], [29]. Here, we exploited large-scale RNAi-mediated LOF in cell-based assays to uncover the biological relevance of the NAT family of RNAs. We report the construction of a comprehensive siRNA library for functional analysis of NATs and confirm that seven antisense transcripts impact the human cell viability phenotype.

## Results

### Design of siRNA library

The natural antisense transcripts examined in this study comprise most of the nearly 1,000 sense-antisense transcript pairs, conserved between human and mouse [16], originally identified through the FANTOM3 transcriptomics effort. We used siSearch [30] to rank all possible siRNAs targeting antisense transcripts of the ∼1000 NAT pairs. We then selected high scoring siRNAs and tested these for specificity, using a wu-blast search against the FANTOM3 transcriptome dataset. Those highly ranked, specific siRNAs meeting selection criteria were selected for each NAT, resulting in 2000 siRNA oligos targeting 797 NATs (172 coding and 625 non-protein coding NATs). For coding-coding pairs, one gene was arbitrarily assigned as NAT, although the other transcript could also be considered the antisense transcript. In most cases we tested three individual siRNA molecules for each NAT to minimize non-specific results. Each siRNA was designed to target only the non-overlapping part of the antisense RNA and each siRNA was designed to have less binding affinity in the 5’ of the negative strand [31]. The sequences for all siRNAs used in the RNAi screen are listed in Table-S1.

### Cell viability and proliferation screen

**Figure 1:**
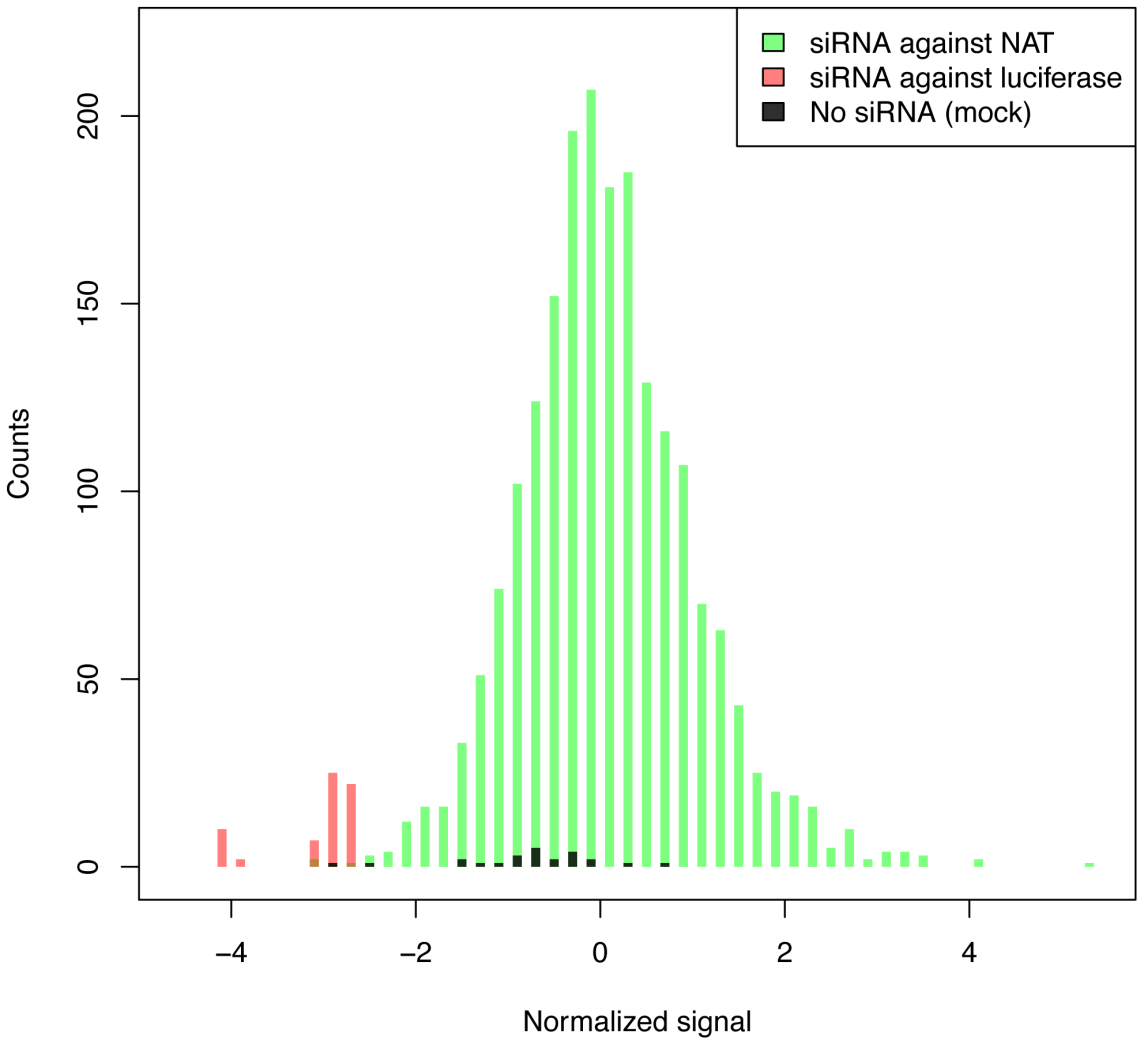
Histograms of normalized luciferase intensity for samples and controls. Intensities were normalized to the plate median (see Methods) so that extreme positive values indicate an increase in viability relative to the median and extreme negative signals indicate a decrease in viability.

We used the Multidrop 384Titan Robot, designed to minimize human experimental errors, to perform large-scale cell viability screening on HEK293T cells in 384-well microtiter plates. Cells were plated and co-transfected with pGL3 luciferase vector and individual siRNAs and after 48 hours, luciferase activity was measured as an indicator of cell viability (Table-S2). Each plate included 8 wells of siRNA for pGL3 RNA as a positive control and 8 wells with no siRNA (mock transfection) as a negative control (a detailed protocol description can be found in the methods section). Figure 1 shows the distributions of luciferase intensity, normalized and averaged over two replicate experiments, for NAT-targeted siRNA and control measurements. Knockdown of luciferase (red bars) consistently resulted in very low signal, confirming transfection and knockdown efficiency in the system. Signals for mock transfection (black bars) were on average somewhat lower than signals from wells with experimental siRNA (green bars), possibly due to toxicity of the transfection reagents in the absence of siRNA. A ranked result list, including normalized signals for all targeted transcripts, is given in Table-S3. The signals were approximately normally distributed (Figure S1).

### Validation of cell viability phenotype

**Figure 2:**
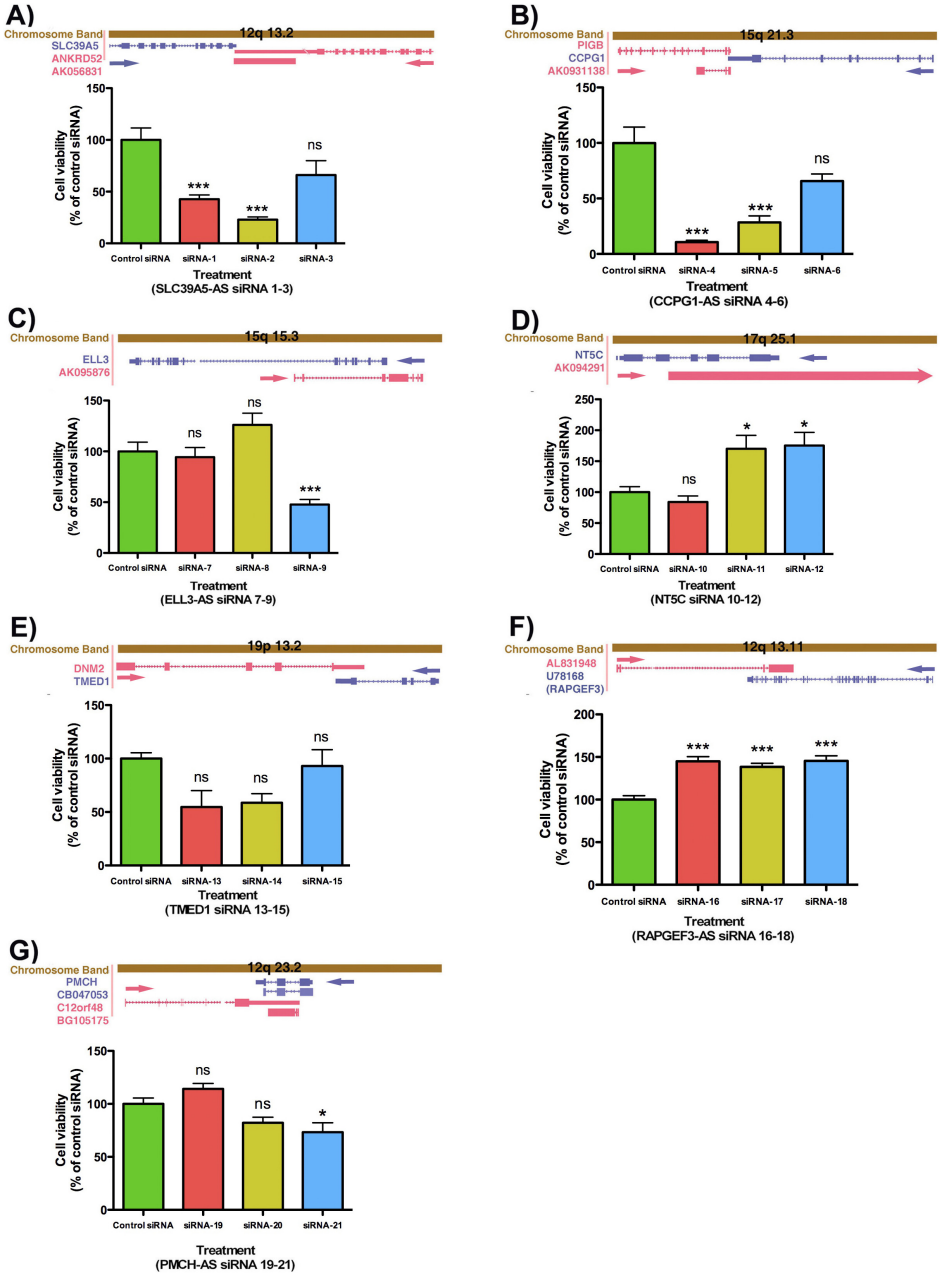
Validation of cell viability screen. A–G) targets were selected based on their Z-score and the number of effective siRNA for the same target in the hit list (Table-S3). A schematic with the genomic organization for each NAT, drawn to scale, is indicated as an inset for each of the seven prioritized targets. In the genomic schematics, antisense sequences are listed in red, sense sequences are listed in blue, and the arrows indicate transcriptional direction. Boxes are indicating exons and lines are indicating introns. Four out of seven targets were validated as follows: siRNAs for solute carrier family 39 (metal ion transporter), member 5- antisense (SLC39A5-AS), cell cycle progression 1- antisense (CCPG1-AS), 5',3'-nucleotidase, cytosolic- antisense (NT5C-AS), RAP guanine-nucleotide-exchange factor 3- antisense (RAPGEF3-AS) were able to reduce or increase luciferase activity compared to a control siRNA, consistent with the original cell viability screen. siRNAs for elongation factor RNAse II-like 3- antisense (ELL3-AS), interleukin 1 receptor-like 1 ligand precursor- antisense (TMED1-AS) and pro-melanin-concentrating hormone- antisense (PMCH-AS) were not able to reproduce the same change in luciferase activity as compared to the original cell viability screen. There were 6 biological repeats for each treatment. The data presented in graphs as a comparison with control siRNA-treated groups. We calculated the significance of each treatment as a p value (*** = P<0.0001, * = P<0.05, ns = P>0.05) and depicted in top of each graph.

Large-scale siRNA-mediated analysis of cell viability, like other phenotypes, has the limitation of occasional inconsistency with reproducibility, making validation experiments crucial [32]. We selected 7 targets (Table 1) for validation by considering several factors: the magnitude of the viability change as indicated by normalized luciferase intensity, the number of siRNAs altering the luciferase intensity in the same direction, and any documented involvement of sense transcripts in cell survival and/or proliferation. For each of the 7 selected targets, we re-tested all 3 siRNAs, even though not all of these siRNAs produced a strong change in luciferase intensity in the primary screen. Luciferase intensity was normalized by comparison with plate median in the primary screen and by comparison with a control siRNA in the validation screen (Figure-2). The difference in normalization approach is necessitated by the difference in scale between the screens. The validation results largely agree with the primary screen, indicating that our primary screening results are generally reproducible.

**Table 1:** Selected targets from RNAi screen for validation studies.

Cell viability	Acc #	Sense ID	Sense description	Antisense ID	Antisense description
Downregulation siRNA(1–3)	AK056831	SLC39A5	zinc transporter	ANKRD52	ankyrin repeat domain-containing protein 52
Downregulation siRNA(4–6)	AK093138	CCPG1	cell cycle progression 1 isoform 2	Noncoding	
Downregulation siRNA(7–9)	AK093233	ELL3	elongation factor RNAse II-like 3	Noncoding	
Upregulation siRNA(10–12)	AK094291	NT5C	5',3'-nucleotidase, cytosolic	Noncoding	
Upregulation siRNA(13–15)	AK097967	TMED1	interleukin 1 receptor-like 1 ligand precursor	DNM2	dynamin 2 isoform 2
Upregulation siRNA(16–18)	AL831948	RAPGEF3	RAP guanine-nucleotide-exchange factor 3	Noncoding	
DownregulationsiRNA(19–21)	BG105175	PMCH	pro-melanin-concentrating hormone	C12orf48	hypothetical protein LOC55010

### Validated targets

We set strict criteria for target validation, requiring a significant (P<0.05) change in luciferase intensity compared to control siRNA for at least 2 out of 3 siRNAs. Three of the selected targets are protein-coding genes that overlap other coding genes, whereas the remaining four targets appear to be noncoding RNA (ncRNA). We were able to validate 1 out of 3 protein-coding targets and 3 out of 4 ncRNA targets, for a combined success rate of 57%. Specifically, we confirmed reduction in luciferase activity for siRNAs against the noncoding NAT of cell cycle progression 1 (*CCPG1*) (Figure-2B) and for siRNAs targeted against the gene *ANKRD52*, which encodes a protein of unknown function and overlaps SLC39A5, a metal ion transporter gene (Figure-2A). Further, we confirmed an increase in luciferase activity for siRNAs against noncoding NATs of two genes: the nucleotidase gene *NT5C* (Figure-2D) and the gene *RAPGEF3* (Figure-2F). The latter encodes a guanine-nucleotide-exchange factor (known as Epac1) implicated in the regulation of cell proliferation [33]. Our failure to validate three of the selected targets (*ELL3-AS*, *DNM2* and *C12orf48*; Figure-2 C, E, and G) can in part be explained by our validation criteria being more stringent than our criteria for selecting transcripts for validation. For example, *DNM2* and *C12orf48* only showed a strong signal change for a single siRNA in the primary screen (Table-S3).

### Cell cycle and cell proliferation analysis

**Figure 3:**
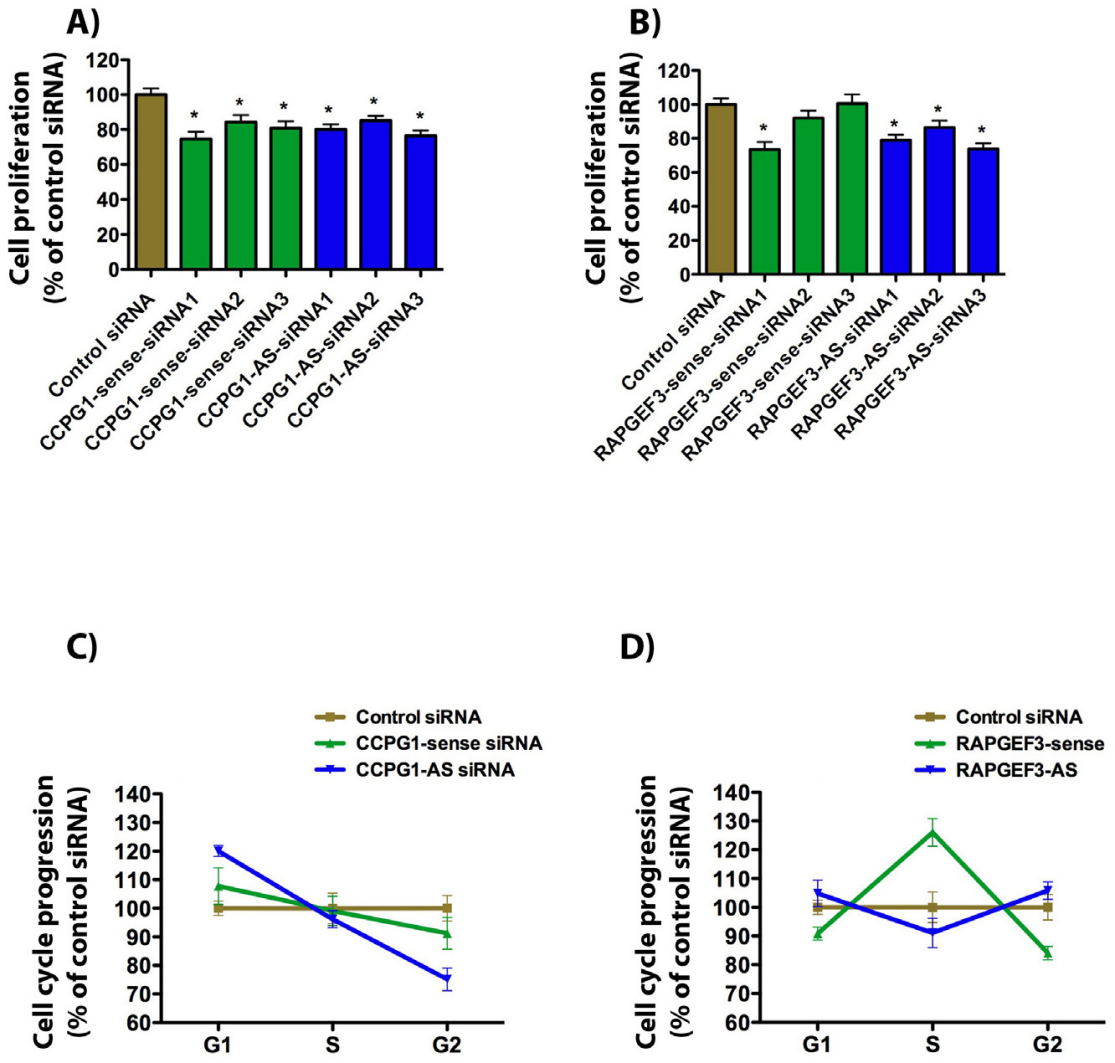
Cell cycle (FACS) and proliferation analysis. Knockdown of CCPG1-AS (A) and RAPGEF3-AS (B) decreases cell proliferation detected with the Cyquant cell proliferation dye. C) CCPG1-AS siRNA, as compared to control siRNA, increases the number of cells in G1 phase and reduces the number of cells in G2 phase as measured by FACS. D) siRNA silencing of RAPGEF3 sense transcript increases the number of cells in S phase and reduces the number of cells in G2 phase. RAPGEF3-AS siRNA produces no significant impact on cell cycle detected by FACS analysis. We calculated the significance of each treatment as a p value and depicted in top of each graph; (* = P<0.05).

Change in cell viability could result from alterations in cell cycle, global transcription, post-transcriptional regulation, or translational efficacy. We measured cell viability with an additional assay, using the Cyquant cell proliferation dye. For these experiments, we focused on the NATs against *CCPG1* and *RAPGEF3*, because of the roles of these genes in regulation of cell proliferation [33]. We observed that siRNA knockdown of *CCPG1-AS* or *CCPG1*-sense significantly reduced cell proliferation detected with the Cyquant assay (Figure-3A). Cells treated with siRNA to *RAPGEF3-AS* also exhibited a decrease in cell proliferation as measured with the Cyquant dye (Figure-3B). To narrow down the source of the observed phenotype, HEK293T cells were transfected with siRNAs against both sense and antisense transcripts of *CCPG1* and *RAPGEF3* and alterations in cell cycle progression were evaluated by fluorescence activated cell sorting (FACS). We found that siRNA-mediated knockdown of *CCPG1-AS* prompted ∼20% increase in G1 phase and decreased cells in G2 by 25% (Figure-3C). A decrease of cells in G2 phase could indicate impaired progression through the cycle and, therefore, explain the observed cell viability reduction. siRNAs targeting *RAPGEF3-AS* (Figure-3D) and other validated targets (data not shown) produced no significant changes in cell cycle progression.

### Regulatory relationship between sense and antisense RNA molecules

**Figure 4:**
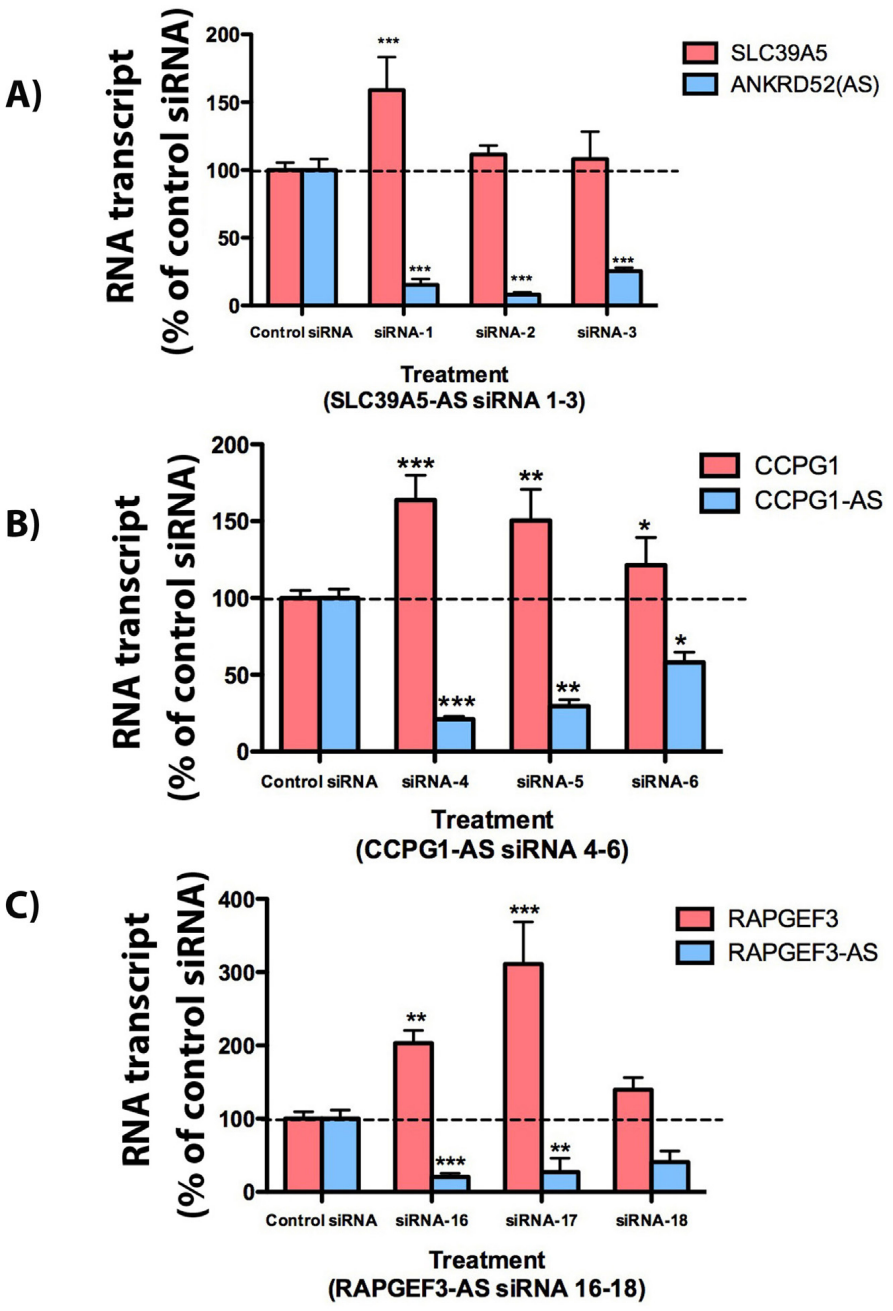
Antisense-mediated regulation of sense mRNA. A) siRNA knockdown of ankyrin repeat domain-containing protein 52 (ANKRD52), the coding NAT for SLC39A5, did not alter the sense mRNA except with modest elevation with one siRNA in HEK293T cells B–C) Knockdown of the noncoding NATs CCPG1-AS and RAPGEF3-AS caused statistically significant up-regulation of the sense mRNA. We calculated the significance of each treatment as a p value and depicted in top of each graph; (** = P<0.001; *** = P<0.0001).

To understand the pattern of antisense mediated regulation of the sense transcript, we selected 3 validated targets with demonstrated sense and antisense RNA expression in HEK293T cells for further analysis using siRNA-mediated knockdown of the antisense transcript (all siRNA and TaqMan primer sequences are listed in Table- S1 and supplementary data-S1). Interestingly, for all three targets we observed evidence for a discordant regulation, where knockdown of the antisense transcript induces an upregulation of the sense RNA. For *ANKRD52*, we only saw this effect with one siRNA (Figure 4A), perhaps indicating that the observed change in cell viability upon *ANKRD52* knockdown is unrelated to any natural antisense interactions it might have with the overlapping *SLC39A5* transcripts. Since *ANKRD52* is a coding gene of unknown function, this may point to a role of the encoded protein in cell viability. For the two other targets, the effect was more robust: knockdown of the noncoding antisense transcripts for *CCPG1* (Figure-4B) and *RAPGEF3* (Figure-4C) led to a 30–80% and 100–250% increase in their corresponding sense mRNA expression, respectively. Additionally, we found that the expression of the antisense transcripts could be downregulated when we knocked down the sense transcripts (Figure-S2). These data suggest a feedback regulatory relationship between the sense and antisense RNA molecules; however, possible mechanisms why this coordinate down-regulation might occur are not known.

### Microarray analysis from RAPGEF-AS knockdown experiments

To examine the global gene expression changes and signaling pathways which could regulate NAT-mediated disruptions in cell viability and proliferation, we carried out microarray gene expression profiling on cells treated with siRNA to RAPGEF3-AS. This approach identifies changes in transcript levels that are directly or indirectly related to RAPGEF3-AS down-regulation or RAPGEF3 up-regulation. We found that 22 protein-coding genes out of 54,675 transcripts represented on the Affymetrix Human U133 Plus 2.0 microarray were either down- or upregulated by greater than 20-fold after siRNA treatment compared to mock transfected controls (Table-S4). Indeed, several of the genes which were found to be dysregulated in the microarray analysis are associated with cell cycle regulatory events, including GDF15 (growth differentiation factor 15) [34], which was about 22.8 fold decreased following RAPGEF3-AS knockdown, and UBE2L3 (ubiquitin-conjugating enzyme E2L 3) [35], which was upregulated by 20.7 fold. A general schematic outlining the biological pathways impacted by RAPGEF3-AS knockdown is illustrated in Figure-S3.

## Discussion

Long ncRNAs present a unique set of challenges for the study of their molecular functions. Unlike microRNAs, whose well described protein interaction pathways direct the processing and utilization of their information content, long ncRNAs still have no common pathway for processing or utilization, leading to persistent doubts about their relevance [36],[37]. Functional validation studies indicate that antisense transcripts are not a uniform group of regulatory RNAs but instead belong to multiple categories with some common features [15]. Several functions have been proposed for NATs including but not limited to transcriptional interference [38], chromatin modifications [24] and processing into small RNAs, which may function as endogenous siRNAs [39], [40], [41]. Often kilobases in length, long ncRNAs appear to contain far more information than could be used efficiently by currently understood pathways, raising the possibility that they could result from spurious transcription [42]. In this study we utilized a screening method to investigate the functional significance of the NAT family of transcripts as a whole. We show that some antisense transcripts are biologically functional by providing evidence that, even in a single cell line, a number of antisense transcripts impact the human cell viability phenotype. Although we only selected seven NATs that scored highly in the screen for follow-up studies, our validation success rate indicates that the screening data likely contains many more examples of NATs associated with cell viability.

Large scale functional screening provides an ideal strategy to probe the significance of NATs [43]. Indeed, Willingham *et al*. adopted this approach to uncover six ncRNAs essential for cell viability, including NRON, a repressor of the transcription factor NFAT [21]. Of note, the viability screen can identify a wide range of sub-cellular phenotypic changes and therefore is a superior method for functional validation studies. Alteration in transcription and translation efficiency as well as changes in cell proliferation and cell cycle can be successfully monitored with a cell viability phenotype. In addition to the original screen, we employed multiple experimental approaches, such as luciferase validation studies, FACS analysis, microarray and Cyquant cell proliferation methods to examine the functional relevance of selected NATs. For instance, we observed that knockdown of *CCPG1-AS* exhibited a significant alteration in cell cycle progression by FACS analysis and that reduction of *RAPGEF3-AS* caused a decrease in cell proliferation as measured with the Cyquant cell proliferation assay. Furthermore, microarray analysis from cells transfected with siRNA against *RAPGEF3-AS* identified several genes that are affected by silencing of noncoding NATs. These changes in gene expression might be explained by knockdown of noncoding antisense transcript or it might be the consequence of sense RAPGEF3 up-regulation. For example, *UBE2L3* (ubiquitin-conjugating enzyme E2L 3), responds robustly to *RAPGEF3-AS* siRNA treatment. One established function of *UBE2L3* is the ubiquitination and subsequent degradation of p53, a well-characterized tumor suppressor gene, which induces cell-cycle arrest or apoptosis in response to cellular stress [35], [44]. Decreased p53 signaling could explain the observed change in luciferase expression after treatment with siRNA to *RAPGEF3-AS*. Indeed, *RAPGEF3-AS* knockdown reduced the transcript levels of p53 almost 4-fold in the microarray analysis. In this context, our data not only reveals a biological role for these NATs, but uncovers a rich texture of regulatory modulation of vital cellular signaling landscapes.

Although we showed a biological role for a number of NATs in the assay of cell viability and proliferation, this assay likely covers only a small region of “phenotype space.” We propose that thousands more examples of long ncRNA molecules may have important functions, which cannot be monitored by our cell viability assay. Additionally, human embryonic kidney cells (HEK293T) cells might not express several of the NATs or their corresponding sense partners. Generally, postnatal brain and gonads are two tissues with relatively high expression of NATs; however, antisense RNAs tend to have higher expression in embryonic tissues. HEK293T cells express a relatively high number of NATs, which might be related to their embryonic source. One major limiting factor with siRNA library screening is the transfection efficacy of targeted cells. Unlike HEK293T, terminally differentiated cells including neurons and other neuronal-like cell types do not typically exhibit optimal transfection efficiencies. Therefore, positive hits from our screening can be selected for detailed functional studies in their relevant cell or tissue types. The application of this NAT-focused siRNA library to additional cellular pathways and phenotypic screens will likely reveal further functional roles for this class of regulatory RNAs.

In some cases, both sense and antisense RNAs are protein coding, making it difficult to assign one transcript as the regulatory RNA transcript. For instance, in the SLC39A5/ANKRD52 pair, both genes are protein coding and there appears to be only a very small (∼40 bp) overlap of the 3'-UTRs of the two transcripts. It is worth nothing that about half of the human or mouse genes exhibit alternative polyadenylation among their transcripts [45]. As a result, each defined 3’ end of a transcript has, on average, 1.3 start sites; conversely, each 5’ end has an average 1.8 corresponding 3’ ends [4], [46]. Therefore, this 40 bp overlapping region could very well expand to a few hundred base pairs of complementary nucleotides. Nevertheless, for coding NATs, there is always a chance that the encoded protein, and not regulatory non-protein-coding properties of the transcripts, might mediate the observed effect on cell viability.

Evolutionary conservation can be highly effective for identifying functional elements in genomes [47]. Thus, we selected the transcripts for our current study from the list of one thousand NATs identified in the FANTOM3 project as conserved between human and mice [16]. However, lack of conservation does not necessarily indicate lack of function [48]. The non-conserved sequences might relate to the emergence of human-specific brain features [49]. Reports of functional primate- or human-specific NATs [50], [51], [52], [53] suggest that many non-conserved NATs may indeed play functional roles in the nervous system [23]. The distinction between conserved and non-conserved transcripts is not always clear-cut, as exemplified by the human accelerated region 1 (HAR1). The HAR1 genomic region exhibits a high level of conservation from chicken to chimpanzee, but has changed rapidly in the human lineage. HAR1 gives rise to multiple antisense-overlapping ncRNAs, one of which is specifically expressed in Cajal-Retzius neurons of the developing neocortex of humans and other primates [49]. Since the NAT class of long RNAs comprises over 5000 known members in humans, most of which are ncRNAs [54], and estimates of total long ncRNA species ranges to over 20,000 transcripts [23], we can expect a widespread potential for human specific regulatory complexity from these ncRNAs in tissues such as the nervous system. Overall, our results now suggest that the conserved members of the NAT family have function. They represent a large reservoir of information potentially useful to regulate cellular processes, and may add yet another dimension to our growing understanding of the functional complexity of mammalian gene loci.

## Methods

### siRNA library

We constructed an siRNA library, containing 2000 siRNA targeting 797 conserved NAT (1-3 siRNA to each). Each siRNA was designed to target the antisense transcript but not the corresponding sense transcript partner, using Karolinska Institute siRNA designing tools [30]. List of siRNA oligos, including siRNA design scoring and the siRNA targets are in supplementary table-S1, energies are calculated as previously described [30].

### Luciferase assay

Using Multidrop 384Titan, 50 ng of pGL3 vector (luciferase vector with SV40 promoter), 50 nM of siRNA and transfection reagents (Lipofectamine 2000 0.2% and OptiMEM) were plated in 384 well plates. Equal numbers of HEK-293T cells (5,000/well) were added to each well and incubated at 37°C for 48 hours. Bright-Glu luciferase reagent was added to each well and incubated at room temperature for 5 minutes. Luciferase activity, as a marker of cell viability, was measured by Analyst GT Multimode Reader.

### Statistical analysis

In analyzing the data, we made use of the R/Bioconductor package cellHTS2 [55]. Spatial effects were identified by plotting the intensities by their position on the plates and putative artifacts were masked out (Table-S2). Intensities were centered at 0 by subtracting plate median and scaled by dividing by plate median absolute deviation (MAD). Only experimental siRNA (non-control) wells were used to compute median and MAD. Finally, normalized signals were averaged over the two replicate experiments.

We performed validation experiments with at least 6 biological and 3–6 technical repeats. The data presented in graphs as a comparison with control-treated groups, after post-hoc test of treatment factor using main effect in one or two-way analysis of variance (ANOVA). We calculated the significance of each treatment as a p value and depicted in each graph, (*p<0.05*) was considered significant.

### Validation of cell viability assay

Using Multidrop 384Titan, 50 ng of pGL3 vector (luciferase vector with SV40 promoter), 20 nM of siRNA (three siRNA per target *n = 24*) and transfection reagents (Lipofectamine 2000 0.2% and OptiMEM, Invitrogen, CA) were plated in 96 well plates. Equal number of cells (10,000 per well) were added to each well and incubated at 37°C for 48 hours. Bright-Glo luciferase reagent (Promega, Madison, WI) was added to each well and incubated at room temperature for 5 minutes. Luciferase activity, as a marker of cell viability, was measured by Analyst GT Multimode Reader (Molecular Devices, Sunnyvale, CA) and plotted against control siRNA.

### Cell culture, siRNA transfection and RNA isolation

HEK-293T cells were cultured in MEM plus 10% FBS. Cells in the logarithmic growth phase were transfected with 20 nM of siRNA using 0.2% Lipofectamine 2000 according to manufacturer's instructions (Invitrogen, Carlsbad, CA). Cells were incubated for 48 hours prior to RNA isolation. Total RNA was extracted using RNeasy mini kit (QIAGEN, Valencia, CA). All samples were treated with RNAse-free DNase (QIAGEN, Valencia, CA) for 15 minutes as described in the manufacturer's protocol. RNA concentrations were measured using the NanoDrop® ND-1000 UV-Vis Spectrophotometer. Equal amounts of RNA were reverse transcribed using TaqMan reverse transcription reagents (Applied Biosystems, Foster City, CA) according to the manufacturer's protocol.

### Microarray analysis

HEK293T cells were treated with 20 nM of control siRNA (Ambion) or siRNA to RAPGEF3-AS. After 48 hours post-transfection, RNA was isolated from the cells as outlined **in** the cell culture section above and pooled from 6 replicates each for control and RAPGEF3-AS siRNA treated samples. Double-stranded cDNA was prepared from 1 µg of total RNA using the Affymetrix cDNA synthesis kit and then *in vitro* transcribed using an IVT labeling kit (Affymetrix), with the cRNA product purified using a GeneChip Sample Cleanup Module (Affymetrix). 20 µg biotin-labeled cRNA was fragmented and hybridized to Affymetrix Human U133 Plus 2.0 microarray overnight in the Affy 640 hybridization oven with a speed of 60 rpm for 16 hr. Microarrays were washed and stained using the Affymetrix Fluidics Station FS400. GeneChip arrays were scanned using a GeneChip Scanner 3000 (Affymetrix). The probe set intensities were quantified using the GeneChip Operating Software (GCOS) and analyzed with GCRMA normalization using Array Assist Software (Stratagene, La Jolla, CA). All hybridized chips met standard quality control criteria, and mean fluorescence values of each array were scaled to a mean intensity of 500. Microarray data is MIAME compliant and the raw data has been deposited in a MIAME compliant database. We expect to receive accession numbers whilst the manuscript is under review.

### Real-Time PCR

Real-Time PCR (RT-PCR) was carried out with the GeneAmp 7900 (Applied Biosystems, Foster City, CA). The PCR reactions contained 20–40 ng cDNA, Universal Mastermix (Applied Biosystems, Foster City, CA), 300 nM of forward and reverse primers, and 200 nM of probe in a final reaction volume of 15 µl. The primers and probe were designed using File-Builder software (Applied Biosystems, Foster City, CA). The PCR conditions were as follows: 50 C for 2 min then 95 C for 10 min then 40 cycles of 95 C for 15 s and 60 C for 1 min. The results are based on cycle threshold (Ct) values. Differences between the Ct values for experimental and reference genes (18S rRNA) were calculated as ΔΔCt.

### Cyquant cell proliferation assay

HEK293**T** cells were seeded with either 200 cells/well or 1000 cells/well in a 96-well plate and reverse transfected with 20 nM of siRNA for 48 hours. After 48 hours, the media was removed and 100 µls of a diluted Cyquant solution was added to each well. Each plate was then incubated at 37° for one hour and then read on the Fluoroskan Ascent FL from Thermo Corporation.

### Cell cycle analysis

Selected viability-related NATs were subjected to cell cycle analysis. We knocked down NATs using three siRNA for each target, and we used a control negative siRNA (triplicate for each treatment) to examine the effects of selected NATs on the cell cycle. At 48 hours post transfection, we prepared the cells for flow cytometry as follow**s**: cells were washed with PBS, trypsinized and centrifuged at 1,000 rpm for 10 minutes. Then the cells were washed again with PBS before being fixed with 70% ethanol at −2°C overnight. The next day the cells were centrifuged, washed with PBS and re-suspended in 38 mM sodium citrate, 69 µM propidium iodide and 19 µg/ml RNAse A for flow cytometry analysis. Results were analyzed using FlowJo analysis software.

## Supporting Information

Table S1 Library siRNA sequences. Sequences for all siRNAs used in the RNAi screen are listed.
(0.62 MB XLS)

Table S2 Raw data from the RNAi screen. Luciferase intensity prior to normalization for each well on the microtiter plates.
(0.30 MB XLS)

Table S3 Normalized data from the RNAi screen. Normalized luciferase intensities are listed for each target along with some metrics used for ranking the targets. See legend on the second sheet of the Excel file for details.
(0.25 MB XLS)

Table S4 Microarray data. The mean fluorescence values for all transcripts altered greater than 20-fold after siRNA treatment against RAPGEF3 on the Affymetrix array is indicated.
(0.02 MB XLS)

Figure S1 Quantile-quantile plot of normalized luciferase signals, demonstrating that the signals are approximately normally distributed. Signals from control wells are not shown.
(1.49 MB TIF)

Figure S2 Knockdown of sense transcripts. Two to three different siRNAs were used to knockdown the sense transcripts for three of the validated NAT targets. Expression of the sense and NAT mRNA was evaluated by real-time PCR. We calculated the significance of each treatment as a p value and depicted on top of each graph; (** = P<0.001; *** = P<0.0001).
(4.97 MB TIF)

Figure S3 Microarray analysis RNA was extracted from HEK293T cells transfected with siRNA to RAPGEF3-AS and analyzed by Affymetrix array for global gene expression changes. Out of 54,675 transcripts represented on the microarray, 22 genes were altered by greater than 20 fold with siRNA knockdown. The major biological pathways impacted by silencing of RAPGEF3-AS, including cell survival and proliferation, are represented in the schematic generated with Pathway Studio software [56].
(8.92 MB TIF)

## References

[1] Frith MC, Bailey TL, Kasukawa T, Mignone F, Kummerfeld SK (2006). Discrimination of non-protein-coding transcripts from protein-coding mRNA.. RNA Biol.

[2] Hayashizaki Y, Carninci P (2006). Genome Network and FANTOM3: assessing the complexity of the transcriptome.. PLoS Genet.

[3] Katayama S, Tomaru Y, Kasukawa T, Waki K, Nakanishi M (2005). Antisense transcription in the mammalian transcriptome.. Science.

[4] Carninci P, Kasukawa T, Katayama S, Gough J, Frith MC (2005). The transcriptional landscape of the mammalian genome.. Science.

[5] Cheng J, Kapranov P, Drenkow J, Dike S, Brubaker S (2005). Transcriptional maps of 10 human chromosomes at 5-nucleotide resolution.. Science.

[6] Willingham AT, Dike S, Cheng J, Manak JR, Bell I (2006). Transcriptional landscape of the human and fly genomes: nonlinear and multifunctional modular model of transcriptomes.. Cold Spring Harb Symp Quant Biol.

[7] Amaral PP, Mattick JS (2008). Noncoding RNA in development.. Mamm Genome.

[8] Kapranov P, Cawley SE, Drenkow J, Bekiranov S, Strausberg RL (2002). Large-scale transcriptional activity in chromosomes 21 and 22.. Science.

[9] Kapranov P, Cheng J, Dike S, Nix DA, Duttagupta R (2007). RNA maps reveal new RNA classes and a possible function for pervasive transcription.. Science.

[10] Cawley S, Bekiranov S, Ng HH, Kapranov P, Sekinger EA (2004). Unbiased mapping of transcription factor binding sites along human chromosomes 21 and 22 points to widespread regulation of noncoding RNAs.. Cell.

[11] Yelin R, Dahary D, Sorek R, Levanon EY, Goldstein O (2003). Widespread occurrence of antisense transcription in the human genome.. Nat Biotechnol.

[12] Shendure J, Church GM (2002). Computational discovery of sense-antisense transcription in the human and mouse genomes.. Genome Biol.

[13] Fahey ME, Moore TF, Higgins DG (2002). Overlapping antisense transcription in the human genome.. Comp Funct Genomics.

[14] Lehner B, Williams G, Campbell RD, Sanderson CM (2002). Antisense transcripts in the human genome.. Trends Genet.

[15] Faghihi MA, Wahlestedt C (2009). Regulatory roles of natural antisense transcripts.. Nat Rev Mol Cell Biol.

[16] Engstrom PG, Suzuki H, Ninomiya N, Akalin A, Sessa L (2006). Complex Loci in human and mouse genomes.. PLoS Genet.

[17] St Laurent G, Wahlestedt C (2007). Noncoding RNAs: couplers of analog and digital information in nervous system function?. Trends Neurosci.

[18] Faghihi MA, Modarresi F, Khalil AM, Wood DE, Sahagan BG (2008). Expression of a noncoding RNA is elevated in Alzheimer's disease and drives rapid feed-forward regulation of beta-secretase.. Nat Med.

[19] Jolly C, Lakhotia SC (2006). Human sat III and Drosophila hsr omega transcripts: a common paradigm for regulation of nuclear RNA processing in stressed cells.. Nucleic Acids Res.

[20] Rossignol F, Vache C, Clottes E (2002). Natural antisense transcripts of hypoxia-inducible factor 1alpha are detected in different normal and tumour human tissues.. Gene.

[21] Willingham AT, Orth AP, Batalov S, Peters EC, Wen BG (2005). A strategy for probing the function of noncoding RNAs finds a repressor of NFAT.. Science.

[22] Lein ES, Hawrylycz MJ, Ao N, Ayres M, Bensinger A (2007). Genome-wide atlas of gene expression in the adult mouse brain.. Nature.

[23] Mercer TR, Dinger ME, Sunkin SM, Mehler MF, Mattick JS (2008). Specific expression of long noncoding RNAs in the mouse brain.. Proc Natl Acad Sci U S A.

[24] Yu W, Gius D, Onyango P, Muldoon-Jacobs K, Karp J (2008). Epigenetic silencing of tumour suppressor gene p15 by its antisense RNA.. Nature.

[25] Swiezewski S, Liu F, Magusin A, Dean C (2009). Cold-induced silencing by long antisense transcripts of an Arabidopsis Polycomb target.. Nature.

[26] Kittler R, Pelletier L, Heninger AK, Slabicki M, Theis M (2007). Genome-scale RNAi profiling of cell division in human tissue culture cells.. Nat Cell Biol.

[27] Willingham AT, Deveraux QL, Hampton GM, Aza-Blanc P (2004). RNAi and HTS: exploring cancer by systematic loss-of-function.. Oncogene.

[28] Brass AL, Dykxhoorn DM, Benita Y, Yan N, Engelman A (2008). Identification of host proteins required for HIV infection through a functional genomic screen.. Science.

[29] Kassner PD (2008). Discovery of novel targets with high throughput RNA interference screening.. Comb Chem High Throughput Screen.

[30] Chalk AM, Wahlestedt C, Sonnhammer EL (2004). Improved and automated prediction of effective siRNA.. Biochem Biophys Res Commun.

[31] Schwarz DS, Hutvagner G, Du T, Xu Z, Aronin N (2003). Asymmetry in the assembly of the RNAi enzyme complex.. Cell.

[32] Echeverri CJ, Beachy PA, Baum B, Boutros M, Buchholz F (2006). Minimizing the risk of reporting false positives in large-scale RNAi screens.. Nat Methods.

[33] Misra UK, Pizzo SV (2009). Epac1-induced cellular proliferation in prostate cancer cells is mediated by B-Raf/ERK and mTOR signaling cascades.. J Cell Biochem.

[34] Chang JT, Chan SH, Lin CY, Lin TY, Wang HM (2007). Differentially expressed genes in radioresistant nasopharyngeal cancer cells: gp96 and GDF15.. Mol Cancer Ther.

[35] Moynihan TP, Cole CG, Dunham I, O'Neil L, Markham AF (1998). Fine-mapping, genomic organization, and transcript analysis of the human ubiquitin-conjugating enzyme gene UBE2L3.. Genomics.

[36] Wang J, Zhang J, Zheng H, Li J, Liu D (2004). Mouse transcriptome: neutral evolution of 'non-coding' complementary DNAs.. Nature.

[37] Ponjavic J, Ponting CP, Lunter G (2007). Functionality or transcriptional noise? Evidence for selection within long noncoding RNAs.. Genome Res.

[38] Osato N, Suzuki Y, Ikeo K, Gojobori T (2007). Transcriptional interferences in cis natural antisense transcripts of humans and mice.. Genetics.

[39] Watanabe T, Totoki Y, Toyoda A, Kaneda M, Kuramochi-Miyagawa S (2008). Endogenous siRNAs from naturally formed dsRNAs regulate transcripts in mouse oocytes.. Nature.

[40] Okamura K, Balla S, Martin R, Liu N, Lai EC (2008). Two distinct mechanisms generate endogenous siRNAs from bidirectional transcription in Drosophila melanogaster.. Nat Struct Mol Biol.

[41] Okamura K, Lai EC (2008). Endogenous small interfering RNAs in animals.. Nat Rev Mol Cell Biol.

[42] Amaral PP, Dinger ME, Mercer TR, Mattick JS (2008). The eukaryotic genome as an RNA machine.. Science.

[43] Mattick JS (2005). The functional genomics of noncoding RNA.. Science.

[44] Riley T, Sontag E, Chen P, Levine A (2008). Transcriptional control of human p53-regulated genes.. Nat Rev Mol Cell Biol.

[45] Tian B, Hu J, Zhang H, Lutz CS (2005). A large-scale analysis of mRNA polyadenylation of human and mouse genes.. Nucleic Acids Res.

[46] Ponting CP, Oliver PL, Reik W (2009). Evolution and functions of long noncoding RNAs.. Cell.

[47] Siepel A, Bejerano G, Pedersen JS, Hinrichs AS, Hou M (2005). Evolutionarily conserved elements in vertebrate, insect, worm, and yeast genomes.. Genome Res.

[48] Pang KC, Frith MC, Mattick JS (2006). Rapid evolution of noncoding RNAs: lack of conservation does not mean lack of function.. Trends Genet.

[49] Pollard KS, Salama SR, Lambert N, Lambot MA, Coppens S (2006). An RNA gene expressed during cortical development evolved rapidly in humans.. Nature.

[50] Lipovich L, Vanisri RR, Kong SL, Lin CY, Liu ET (2006). Primate-specific endogenous cis-antisense transcription in the human 5q31 protocadherin gene cluster.. J Mol Evol.

[51] Khalil AM, Faghihi MA, Modarresi F, Brothers SP, Wahlestedt C (2008). A novel RNA transcript with antiapoptotic function is silenced in fragile x syndrome.. PLoS ONE.

[52] Liu QR, Lu L, Zhu XG, Gong JP, Shaham Y (2006). Rodent BDNF genes, novel promoters, novel splice variants, and regulation by cocaine.. Brain Res.

[53] Pruunsild P, Kazantseva A, Aid T, Palm K, Timmusk T (2007). Dissecting the human BDNF locus: bidirectional transcription, complex splicing, and multiple promoters.. Genomics.

[54] Yin Y, Zhao Y, Wang J, Liu C, Chen S (2007). antiCODE: a natural sense-antisense transcripts database.. BMC Bioinformatics.

[55] Boutros M, Bras LP, Huber W (2006). Analysis of cell-based RNAi screens.. Genome Biol.

[56] Suderman M, Hallett M (2007). Tools for visually exploring biological networks.. Bioinformatics.

