# Supplementary figures and images for: RNAi Screen Indicates Widespread Biological Function for Human Natural Antisense Transcripts
Source: PLoS One. 2010 Oct 4;5(10):e13177. doi: 10.1371/journal.pone.0013177 (PMC2949395; doi:10.1371/journal.pone.0013177)

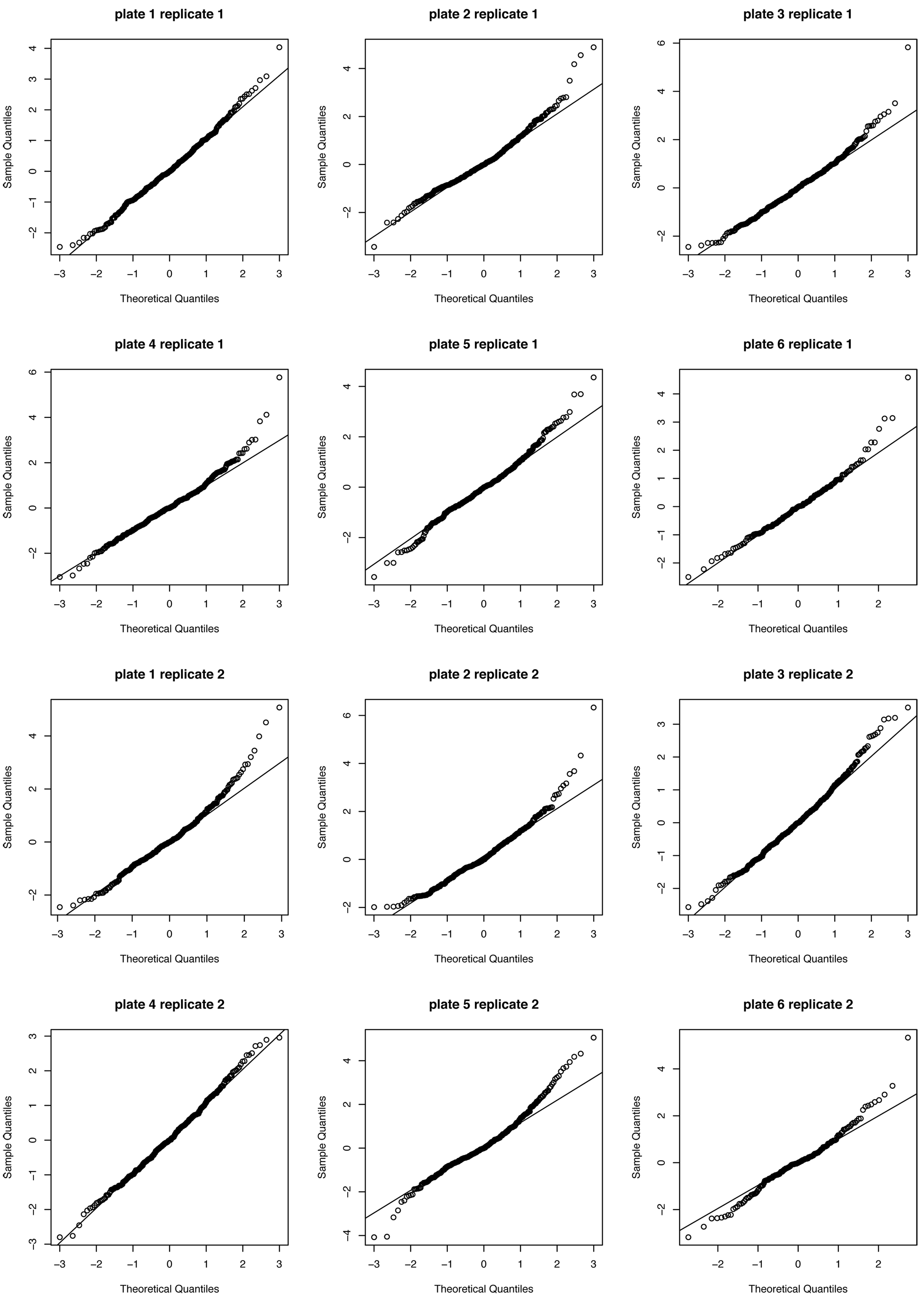

Supplement: Figure S1 — Quantile-quantile plot of normalized luciferase signals, demonstrating that the signals are approximately normally distributed. Signals from control wells are not shown. (1.49 MB TIF) [file pone.0013177.s005.tif]

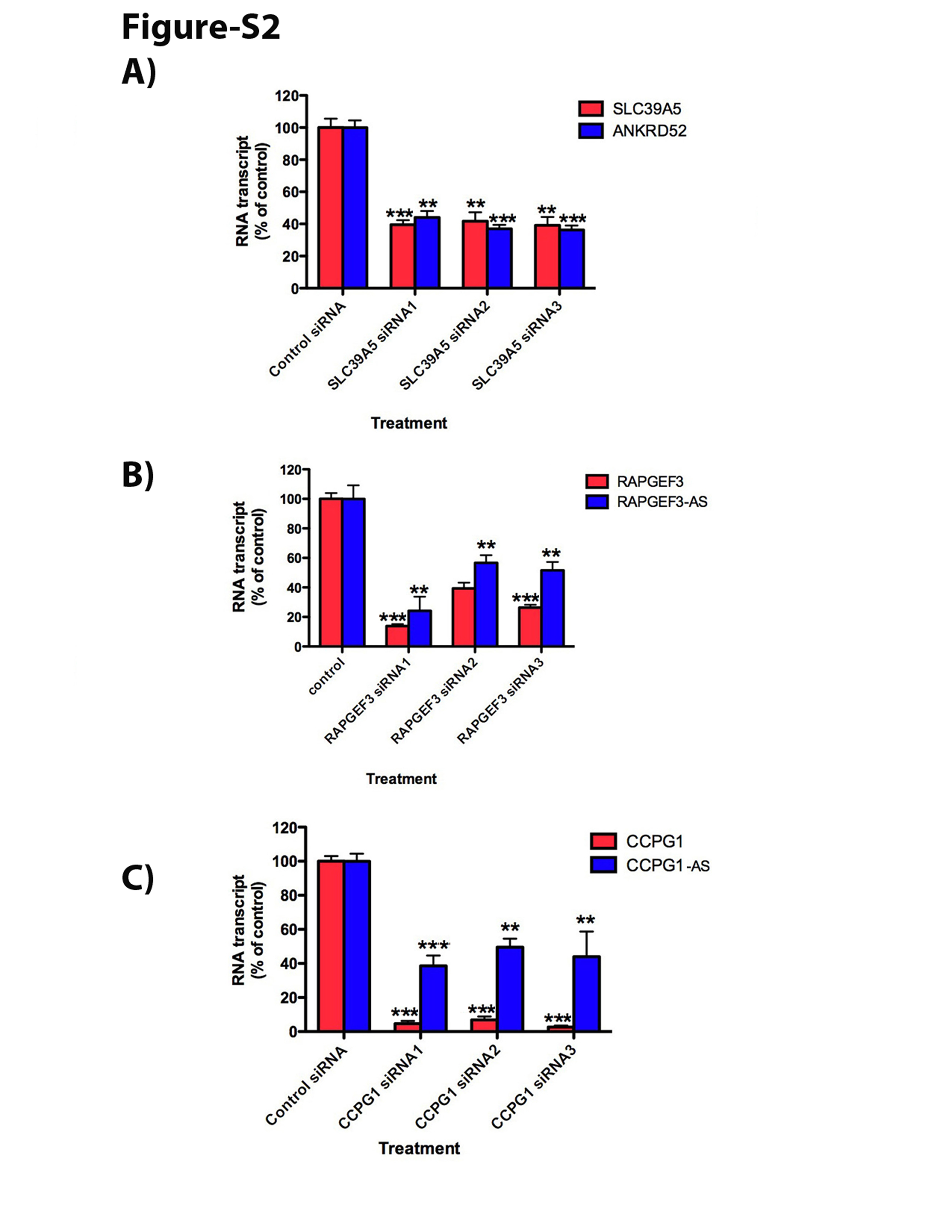

Supplement: Figure S2 — Knockdown of sense transcripts. Two to three different siRNAs were used to knockdown the sense transcripts for three of the validated NAT targets. Expression of the sense and NAT mRNA was evaluated by real-time PCR. We calculated the significance of each treatment as a p value and depicted on top of each graph; (** = P<0.001; *** = P<0.0001). (4.97 MB TIF) [file pone.0013177.s006.tif]

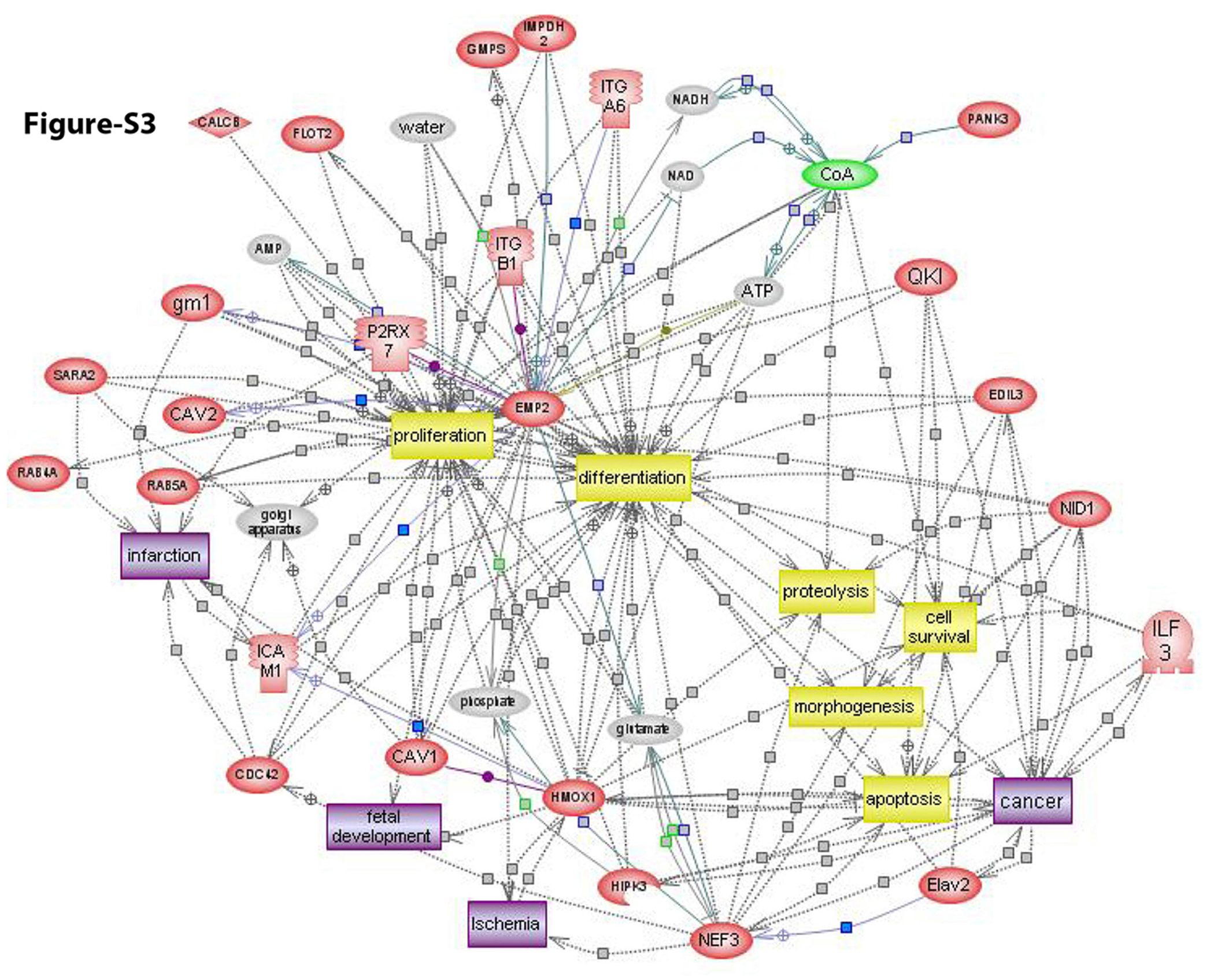

Supplement: Figure S3 — Microarray analysis RNA was extracted from HEK293T cells transfected with siRNA to RAPGEF3-AS and analyzed by Affymetrix array for global gene expression changes. Out of 54,675 transcripts represented on the microarray, 22 genes were altered by greater than 20 fold with siRNA knockdown. The major biological pathways impacted by silencing of RAPGEF3-AS, including cell survival and proliferation, are represented in the schematic generated with Pathway Studio software [56]. (8.92 MB TIF) [file pone.0013177.s007.tif]
